# The Association of Weather Variability and Under Five Malaria Mortality in KEMRI/CDC HDSS in Western Kenya 2003 to 2008: A Time Series Analysis

**DOI:** 10.3390/ijerph120201983

**Published:** 2015-02-10

**Authors:** Maquins Sewe, Joacim Rocklöv, John Williamson, Mary Hamel, Amek Nyaguara, Frank Odhiambo, Kayla Laserson

**Affiliations:** 1 KEMRI Centre for Global Health Research, Kisumu, Kenya, Box 1578, Kisumu 40100, Kenya; E-Mails: namek@kemricdc.org (A.N.); fodhiambo@kemricdc.org (F.O.); 2 Department of Public Health and Clinical Medicine, Epidemiology and Global Health, Umeå University, Umeå SE-901 85, Sweden; E-Mail: joacim.rocklov@envmed.umu.se; 3 Centers for Disease Control and Prevention, Centre for Global Health, 600 Clifton Rd, Atlanta, GA 30333, USA; E-Mails: jow5@cdc.gov (J.W.); mlh8@cdc.gov (M.H.); kel4@cdc.gov (K.L.)

**Keywords:** malaria mortality, KEMRI/CDC HDSS, general additive model, rainfall, temperature, lag, Kenya

## Abstract

Malaria is among the leading causes of mortality in the younger under-five group of children zero to four years of age. This study aims at describing the relationship between rainfall and temperature on under-five malaria or anaemia mortality in Kenya Medical Research Institute and United States Centers for Disease Control (KEMRI/CDC) Health and Demographic Surveillance System (HDSS). This study was conducted through the ongoing KEMRI and CDC collaboration. A general additive model with a Poisson link function was fit to model the weekly association of lagged cumulative rainfall and average temperature on malaria/anemia mortality in KEMRI/CDC HDSS for the period 2003 to 2008. A trend function was included in the model to control for time trends and seasonality not explained by weather fluctuations. 95% confidence intervals was presented with estimates. Malaria or anemia mortality was found to be associated with changes in temperature and rainfall in the KEMRI HDSS, with a delay up to 16 weeks. The empirical estimates of associations describe established biological relationships well. This information, and particularly, the strength of the relationships over longer lead times can highlight the possibility of developing a predictive forecast with lead times up to 16 weeks in order to enhance preparedness to high transmission episodes.

## 1. Introduction

Malaria is one of the leading causes of mortality among the under-five population with over 86% of the overall malaria mortality occurring in this age group [1]. The WHO reported over 655,000 malaria deaths in the world in 2010, 90% of which occurred in Africa [1]. In Kenya, over 26,000 deaths were attributable to malaria in 2010 [1]. Approximately 40% of the Kenyan population is at high risk of malaria infection and most of the cases of malaria infection in Kenya are due to infection with the *Plasmodium falciparum* parasite [1]. According to the KEMRI/CDC HDSS 2008 annual report, 31% of the total deaths in Asembo, Gem and Karemo among the under-five population (excluding neonates) were due to malaria, and over 87% of pediatric admissions at Siaya District hospital were due to malaria [2].

Human malaria parasites are transmitted by mosquitoes of the genus anopheles; the most efficient are *Anopheles gambiae* and *Anopheles arabiensis* [3]. The most common malaria mosquito vectors *i*n KEMRI/CDC HDSS study area are *An. gambiae* ss and *funestus* [4], but for many years the primary vector has been *arabiensis* [5].

Several studies [6,7,8,9] have been conducted to illuminate the effect of weather factors, mainly temperature and rainfall, on malaria vector proliferation. The mosquito vectors of malaria parasites are sensitive to changes in climate. Climatic variables such as rainfall and temperature are known to determine mosquito reproduction and mortality [10].

A mathematical model exploring the relationship of temperature and progeny of malaria parasite in mosquitoes showed that at a temperature of 20 °C and 25 °C it would take between 22–23 days and 12–14 days, respectively, for the sporogony of *P. falciparum.*

Research has estimated the mean temperature ideal for the development of mosquito vectors to be 25–27 °C while the development terminates at 10 °C, and at 40 °C when vector survival rate is low. Rainfall affects vector abundance by providing breading sites for vectors and supporting vector development during the immature stages [11]. Continuous rains result in flooding which in turn clears mosquito breeding sites, while intermittent rain with long spells of sunshine provide a suitable environment for mosquito vector proliferation [12].

Other studies have shown that the most suitable temperature conditions for mosquito larvae survival is in the range of 20–30 °C [13,14]. The annual variability of malaria infection in the highlands of Kenya is greatly influenced by climate events such as El Niño with epidemic outbreaks of malaria observed during such events [15,16]. A study conducted in Kisumu, Kenya showed a 22% increase in the prevalence of malaria parasites after a period of extensive rainfall [17]. It has been shown that the population of mosquito vector *An. gambiae* is correlated with lagged rainfall. A study in Miwani in Western Kenya showed that rainfall during the previous week was significantly associated with the population of female *An. gambiae* [18]. Lagged total annual rainfall was shown not to have any impact on malaria/anemia parasitemia in the KEMRI/CDC HDSS between the years 2003 and 2008 [19]. However, one possible explanation for this may be entomological inoculation rates (EIR) being low and stable during this period.

To date few studies have researched the relationship between weekly weather and malaria mortality in Kenya directly, and explored how well the intermittent biological mechanisms can be described and confirmed empirically by routine surveillance data. We seek to determine the effect of rainfall and temperature on under five malaria/anemia mortality in the Asembo and Gem areas of Western Kenya between 2003 and 2008. Anemia mortality was included because research has shown that malaria is one of the greatest risk factor for anemia deaths e.g., a study in Western Kenya reported that anemia was 10 times more often occurring among children with malaria [20], and that *falciparum* malaria was the primary cause of 46% of severe anemia cases in a study in Kenya [21].

## 2. Results

**Figure 1:**
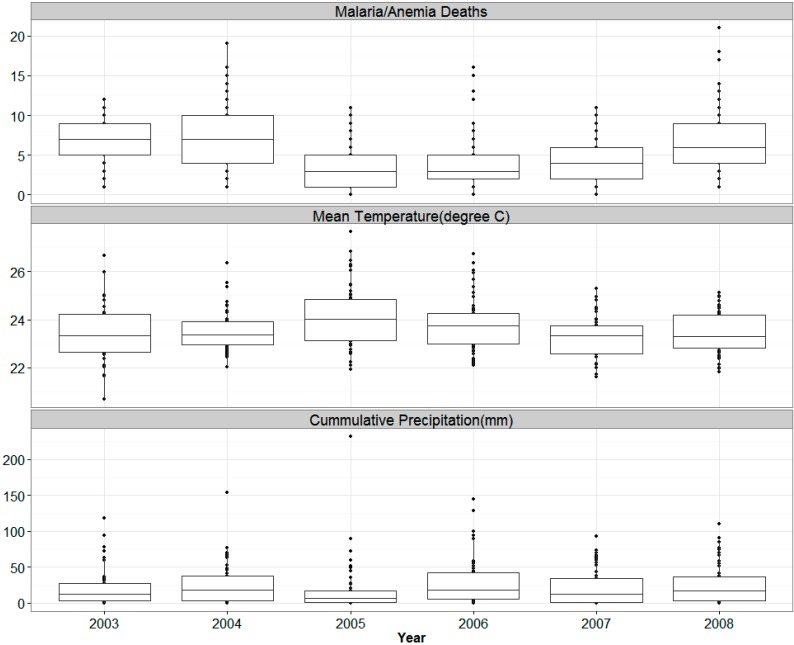
Boxplot of Weekly Malaria/Anemia Deaths among children under five years, Mean temperatures (°C) and cumulative precipitation (mm), 2003–2008.

Table 1 displays univariate statistics of weekly malaria/anemia under five deaths, temperature and rainfall for the years 2003 to 2008 in the KEMRI/CDC HDSS study area. During the period 2003 to 2008 a total of 1768 malaria/anemia deaths were observed among children under the age of five. Figure 1 illustrates the yearly distribution of the deaths. The highest number of deaths was recorded in the year 2004 with 386 deaths. The years 2005 and 2007 showed marked decline by 49% and 41% respectively in the number of malaria/anemia attributable deaths compared to the level in 2004 but there was an upsurge in 2008 almost to the same level observed in 2004. The average weekly number of deaths was 5 with a range of 0 to 21 deaths and the highest average weekly deaths of 7.3 was observed in 2004. In 2003 we observe that over 75% of the weeks had deaths of over 5 cases while we observe extreme number of deaths recorded in certain weeks in 2006 and 2008 with over 15 deaths recorded.

The average weekly mean temperature was 23.6 °C for the period 2003 to 2008 with little variation between the years. The average weekly temperature ranged between 21 °C to 28 °C. Figure 1 shows the distribution of weekly mean temperature for each year of the study in a boxplot. We observe that in the year 2005 over 50% of the weeks had mean temperature of over 24 °C making it the hottest year observed while in 2008 50% of the weeks had mean temperatures of below 23 °C.

The summaries for precipitation are in Table 1. The year 2006 was the wettest, with a total of 1549 mm of precipitation and a mean of 29 mm per week registered while the driest year was 2005 with 970 mm of total rainfall and mean of 18 mm of rainfall per week observed. The weekly total rainfall ranged between 0 to 232 mm. The boxplot in Figure 1 show the distribution of weekly rainfall per year. It is evident that, for example, in 2006 around 75% of the weeks had precipitation while in some other years there was fewer precipitation weeks.

**Table 1:** Summary statistics (weekly) of malaria/anemia deaths among children under five years, mean temperature (°C) and cumulative precipitation (mm) for the period 2003–2008 in Asembo and Gem. Standard deviation (Std) are provided for mean values.

Variable	Summary	Year						
		2003	2004	2005	2006	2007	2008	2003–2008
Malaria/Anemia Deaths	Sum	366	386	195	227	226	368	1768
	Mean (Std *)	6.9(2.7)	7.3(4.3)	3.7(2.8)	4.3(3.8)	4.3(2.6)	6.9(4.5)	5.6(3.8)
	Median	7.0	7.0	3.0	3.0	4.0	6.0	5.0
	Interquartile range	5.0–9.0	4.0–10.0	1.0–5.0	2.0–5.0	2.0–6.0	4.0–9.0	3.0–8.0
	Range	1.0–12.0	1.0–19.0	0.0–11.0	0.0–16.0	0.0–11.0	1.0–21.0	0.0–21.0
Mean Temperature	Mean (Std *)	23.5(1.1)	23.5(0.8)	24.2(1.3)	23.8(1.1)	23.3(0.8)	23.4(0.9)	23.6(1.1)
	Median	23.3	23.4	24.0	23.7	23.3	23.3	23.4
	Interquartile range	22.7–24.2	23.0–23.9	23.1–24.8	23.0–24.2	22.6–23.7	22.8–24.2	22.9–24.2
	Range	20.7–26.6	22.0–26.3	21.9–27.6	22.1–26.7	21.6–25.3	21.8–25.1	20.7–27.6
Cumulative precipitation	Sum	1073	1356	970	1549	1166	1333	7447
	Mean (Std *)	20.2(25.3)	25.6(28.6)	18.3(35.9)	29.2(32.6)	22.0(24.7)	25.2(27.5)	23.4(29.4)
	Median	12.4	18.0	6.9	18.5	12.1	16.8	14.0
	Interquartile range	3.0–26.9	3.0–37.9	0.5–17.2	5.1–41.9	1.5–34.0	3.0–36.1	2.0–33.5
	Range	0.0–118.3	0.0–153.6	0.0–232.2	0.0–144.1	0.0–93.2	0.0–110.0	0.0–232.2

* Standard deviation of the mean values.

**Figure 2:**
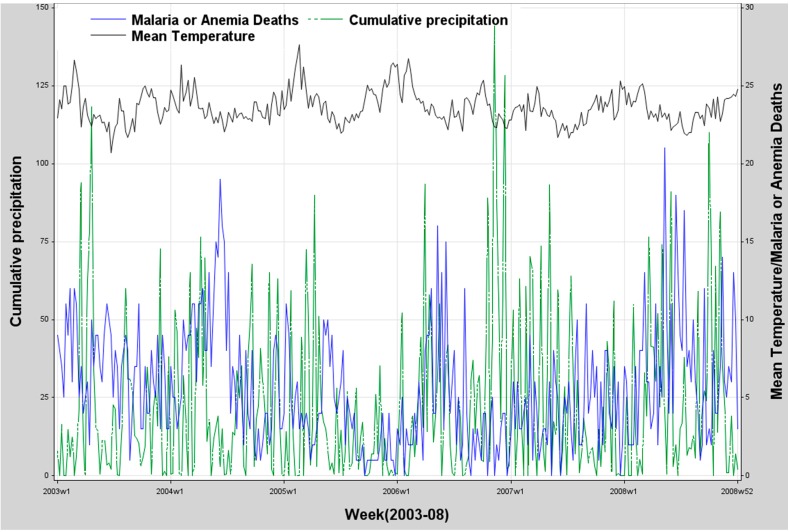
Weekly malaria/anemia deaths among children under five years, mean temperature (°C) and cumulative precipitation (mm) in 2003–2008.

Figure 2 displays the series of malaria/anemia deaths *versus* total weekly rainfall and weekly mean temperatures respectively for the period 2003–2008. We observe consistent seasonal and yearly variation with two peaks in the weather variables, as in the malaria or anemia mortality.

**Figure 3:**
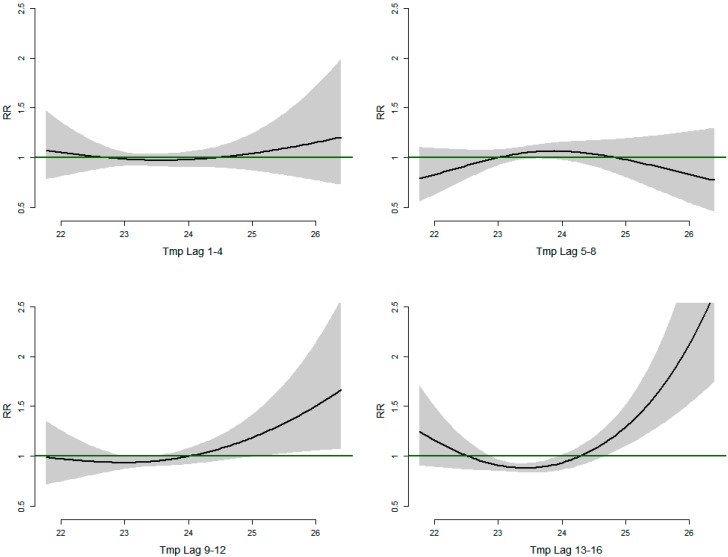
Relative risks of Malaria/Anemia mortality in children under five years with weekly mean temperature (Tmp) at different lag strata.

Figure 3 shows the relative risks of malaria/anemia mortality with different weekly lags of mean temperature. At lags 1–4 weeks we observe a U-shaped relationship between temperature and mortality with mortality declining with low temperature, but increases from 24 °C though we note that it’s not statistically significant as all the confidence intervals for the Relative risks include 1. In the lags 5–8 weeks we see an inverted U-shaped relationship between temperature and mortality. Mortality increasing until 24 °C and then steadily declines. At higher lags 9–12 weeks and 13–16 weeks the temperature mortality relationship exhibits a J-shape with relative risk of mortality increasing linearly with increasing temperatures from 24 °C with greater risk observed in lag strata 13–16 weeks. At temperature below 24 °C the risk of mortality increases in the higher lags from 9 weeks. The malaria-temperature lag relationships show a clearer pattern with longer lag times with the strongest relationship at lag 13–16.

**Figure 4:**
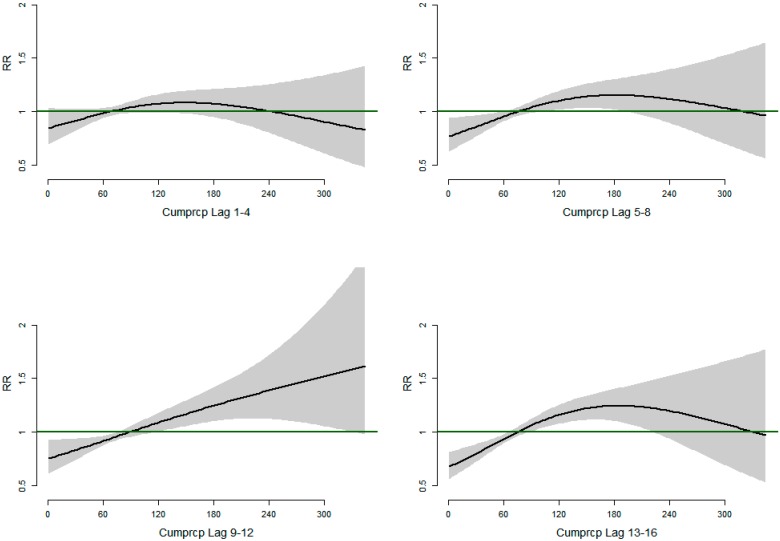
Relative risks of Malaria/Anemia mortality in children under five years with weekly cumulative precipitation at different lag strata.

Figure 4 shows the relative risks of malaria/anemia mortality with different weekly lags of total rainfall. We observe that relative risk of malaria/anemia mortality increases linearly at lags 9–12 weeks with increasing total weekly rainfall. At lags 5–8 and 13–16 weeks, respectively, we observe an inverted U–shaped relationship with increasing risk of mortality for rainfall between 0–180 mm while decreased risk of mortality at rainfall amount of over 180 mm. The association is observed strongest in lag period 9–12 weeks.

**Figure 5:**
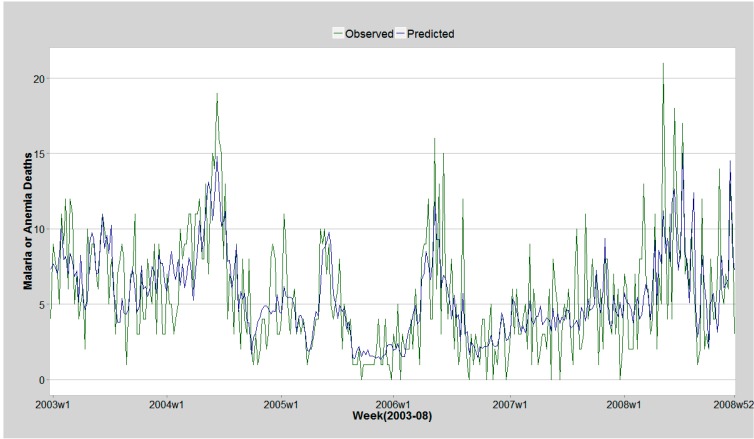
Malaria/Anemia observed deaths and model prediction 2003–2008 Asembo and Gem.

The model selected using backward elimination included cubic spline function of time and lags of temperature and cumulative precipitation from week five onwards. The included auto-regressive terms of previous malaria/anemia deaths improved model fit by clearing autocorrelation in model residuals. The histogram of the model residuals showed they were normally distributed. The model could account for 55% of the variation in weekly malaria/anemia deaths. Comparison of model prediction and observed deaths are illustrated in the Figure 5.

## 3. Discussion

We explored the relationship between rainfall and temperature with malaria and anemia mortality in children under five years of age using a general additive Poisson regression model employing smooth functions. The results depict a delayed effect of weather variables on the malaria/anemia mortality rates. The risk of death due to malaria/anemia among children increased linearly with increasing weekly mean temperatures of over 24 °C 9–12 and 13–16 weeks after higher temperatures begin, while increasing linearly with increasing amount of rainfall 9–12 weeks after rainfall increased. Predictions from the model show a good ability to repeat intra- and inter-annual patterns of malaria deaths in ages under 5 by selected variables.

These findings are consistent with a similar study conducted in Niger that showed that malaria incidence increased by 7% 40 days after rainfall, and that the highest risk was observed between 22 and 39 days with a maximum risk at 33 days [22]. A study in Ghana showed the most significant effect of rainfall on malaria/anemia mortality to be after a lag of nine weeks [23]. This is within the range of our findings. Another study conducted in Ethiopia showed that malaria cases increased linearly with increasing rainfall at lags of six to twelve weeks [24].

The population of the mosquito vector *An. gambiae* has been shown to increase at temperature extremes and a linear increase in vector abundance with increasing rainfall in the hot climatic area of Malindi, Kenya [21]. This study shows similar trends with increasing mortality risk at higher mean weekly temperatures over 24 °C. Our findings also show that the temperature effect on malaria mortality is more delayed than the precipitation effect. The temperature effect is much more pronounced between weeks 13 and 16. These findings portray a biological mechanism underlying mosquito reproduction and malaria incidence and mortality.

We employed general additive Poisson model incorporating smooth functions. This allowed flexibility in modelling effect of temperature and rainfall as non-linear functions. A study in the KEMRI/CDC HDSS concluded that rainfall in the last 3 or 12 months was not associated with risk in community malaria/anemia parasitemia [19]. One possible explanation is that the non-linear relationship between the two parameters was not considered in the modelling (and this would risk canceling out of u-shaped patterns), or that the lag period of 12 months may have been too long to observe any effects. We included a time trend in the model to account for changes in trend in malaria/anemia mortality and to control for all unobserved parameters that could influence malaria/anemia mortality such as social-economic status, prevention and control activities. This was modeled as a cubic time trend function. This function would absorb patterns that were typically inter-annual as would be expected from interventions. However this may not have been adequate to account for all un-measured parameters in the model such as land use that has been shown to influence vector abundance [25]. Previous malaria/anemia deaths up to 16 weeks were included in the model as autoregressive terms to clear autocorrelation remaining in the model residuals. The inclusion of the autoregressive terms improved model ability to predict malaria/anemia cases as depicted in Figure 5 which shows the predicted and observed malaria/anemia deaths. The model explained 55% of the variation in malaria/anemia mortality.

One of the limitation of this study includes the use of mortality data instead of malaria/anemia cases as not all malaria infections result in mortality. A limitations is also the use of Kisumu weather data which is collected approximately 60 km from the KEMRI/CDC HDSS area. Measurements of temperature and rainfall may not be homogeneous due to small variations in altitude and geography and spatial variability between Kisumu and the KEMRI/CDC DSS area. Another limitation of the study is the use of verbal autopsy methodology for defining malaria/anemia deaths, which may lead to misclassification of some of the malaria/anemia cases [26].

## 4. Materials and Methods

### 4.1. Study Setting

KEMRI/CDC HDSS is located in Western Kenya and covers three contiguous regions: Asembo in the Rarieda district, Gem in the Gem district and Karemo in the Siaya district, covering 685 km^2^ with a population of over 220,000. The three regions were enumerated and surveillance began in 2001, 2002 and 2007, respectively. The HDSS continuously collects information on births, deaths, pregnancies, migrations, education, household socio-economic status and morbidity from residents. The basic demographic information is collected every round three times in a year while socio-economic information is collected bi-annually by community interviewers. A round consists of four months. An individual is considered a resident member of the HDSS if he or she was born to a resident mother, enumerated at baseline, or migrated into the study area and stayed for at least four calendar months. The KEMRI/CDC HDSS also collects births and deaths through a separate system using village reporters. This is done to ensure immediate reporting of these events as they occur in the community and to ensure neonatal deaths are captured [27].

### 4.2. Verbal Autopsy

The malaria or anemia causes of death used in this analysis are derived using verbal autopsy methodology. We have combined malaria and anemia deaths because anemia deaths in the KEMRI/CDC HDSS frequently have malaria as the underlying cause [20,21]. Verbal autopsies are conducted on all deaths of HDSS residents and non–residents. The families of the bereaved are allowed one month to mourn after which a verbal autopsy interview is conducted. The VA questionnaires are then given to two clinicians who review the forms and separately specify a probable cause of death. If discordant cause of death are ascribed by the two clinicians, a third clinician reviews the VA questionnaire. If two of the three clinicians fail to agree, an expert panel is constituted to specify the probable cause of death. Cause of death determination using VA is cost effective and reliable in developing settings where real autopsies are not available [28] and has been used in malaria mortality burden estimation [29].

## 5. Meteorological Data

Synoptic weather data derived from the National Oceanic and Atmospheric Administration (NOAA) online data portal was used in this analysis. The meteorological data was monitored as part of the World Meteorological Organization (WMO). We used data for the Kisumu area, which is approximately 60 km from the KEMRI/CDC HDSS study area. We used daily mean surface temperature and 24 hour cumulative precipitation as predictors in the models.

We used data on malaria and anemia deaths among under five population from Asembo and Gem areas of the KEMRI/CDC HDSS and Kisumu rainfall and temperature data for the period 2003 to 2008. The malaria/anemia mortality data was aggregated weekly from the KEMRI/HDSS verbal autopsy data. Weekly mean temperatures and cumulative rainfall were computed from the daily weather data. Lag strata of weekly mean temperature and cumulative rainfall were computed for 1 to 16 weeks. A lag maximum of 16 weeks was chosen taking into account the biological mechanism of malaria transmission and checking cross correlation coefficients between the malaria deaths and the lagged weather variables. The lag strata were grouped as lag 1–4, lag 5–8, lag 9–12 and lag 13–16 corresponding to weeks 1 to 4, weeks 5 to 8, weeks 9 to 12, and weeks 13 to 16, respectively.

## 6. Statistical Analysis

We computed the mean, median, minimum, maximum, range and inter-quartile range of the weather and mortality variables for each year and overall. Two percent of the weather data were missing or considered to be incorrect data input. The missing observations were linearly imputed by neighboring days.

We used Poisson regression with lagged weather variables, allowing for over dispersion, to model the expected number of malaria/anemia deaths for each week. We included a time trend in the model to control for un-observed confounders and to capture changes in malaria/anemia deaths over time not explained by weather variability. Auto regressive terms of malaria/anemia deaths were also included in the model to reduce the remaining auto-correlation of the model residuals. The autoregressive terms were estimated using lags of 1 to 16 weeks of the malaria/anemia deaths. We used smooth cubic regression splines to model both the time trend and the lagged meteorological variables. For the latter we allowed three degrees of freedom for the smooth function while we used 2 degrees of freedom per year for the time trend. The model fit was:

$$\text{log}[E(Y_{t})]=\beta_{0}+\beta_{1}AR[Mal_{t}]+[\overset{4}{\underset{i=1}{\sum }}S[temp_{i},df]+S[cumprcp_{i},df]]+S[trend,df]$$

where $Y_{t}$ ~Poisson, $\beta_{0}\;and\;\beta_{1}$ are parameter estimates; *t* = time in weeks; AR(mal) = auto regressive term of malaria/anemia deaths; s = cubic smoothing function with corresponding degrees of freedom (*df*), $temp_{i}$ = weekly mean temperature at lag strata i, and $cumprcp_{i}$ = weekly cumulative precipitation at lag strata *i*. There were four lag strata corresponding to weeks 1–4, 5–8, 9–12 and 13–16.

We used the Partial Autocorrelation function (PACF) to determine the degrees of freedom for the smoothing function of the time trend. We chose the degrees of freedom that minimized the absolute sum of the PACF of the residuals of the first 10 lags of the model [30]. The degrees of freedom compared were 1, 2, 3, 4 and 5 per year of data in the time series. We used backward elimination to produce the final model. We used data for the period 2003–2007 to initially fit the model while 2008 data were used to test model predictions. We assessed model fit by examining the ACF, PACF, and the cumulative periodogram of the model residuals. We also checked the normality assumption of the residuals by examining normal q-q plots and the plot of the residuals *versus* the predicted values of the response. The analysis was done using MGVC package in R [31].

## 7. Conclusions

This analysis identified lagged patterns of rainfall and temperatures on malaria/anemia mortality in children in KEMRI/CDC HDSS area. The analysis provides more understanding into the timing and the effect of weather parameters on malaria/anemia mortality among children in the KEMRI/CDC HDSS in a way that has not been explored before. The results show predictability of the malaria mortality in relation to weather variability. Further studies should be conducted to estimate the predictiveness at different lag times, as well as spatial heterogeneity.
